# Multimodality Neuromonitoring in Pediatric Neurocritical Care: Review of the Current Resources

**DOI:** 10.7759/cureus.385

**Published:** 2015-11-20

**Authors:** Michael A Galgano, Zulma Tovar-Spinoza

**Affiliations:** 1 Neurosurgery, SUNY Upstate Medical University

**Keywords:** pediatric, brain injury, multimodal monitoring

## Abstract

Brain insults in children represent a daily challenge in neurocritical care. Having a constant grasp on various parameters in the pediatric injured brain may affect the patient’s outcome. Currently, new advances provide clinicians with the ability to utilize several modalities to monitor brain function. This multi-modal approach allows real-time information, leading to faster responses in management and furthermore avoiding secondary insults in the injured brain.

## Introduction and background

In the pediatric population with brain injury, the ideal modality of intracranial monitoring is a topic of debate with very few papers dedicated to this matter. Different etiologies have been cited as a cause for alterations in cerebral pathophysiology in children, such as traumatic brain injury, ischemic events, hydrocephalus, inflammatory encephalitis, spontaneous intraventricular hemorrhage, and intraparenchymal and subarachnoid hemorrhage [1-4]. Aneurysms and arteriovenous malformations, although quite infrequent in this population, do occur and have the potential to cause devastating neurological sequelae [4-7].

Currently, we look at the insulted brain from different perspectives. In this paper, we present a review of the current modalities of monitoring the insulted pediatric brain to gather the best possible information to optimize brain recovery.

Traditionally, the result of a severe intracerebral insult to a child is increased intracranial pressure (ICP). ICP has been the parameter of choice to monitor in children with a low Glasgow Coma Scale (GCS), decreased neurological status, and abnormal neuroradiographic findings. Lumbar puncture (LP) has been historically the most basic method of assessing the intracranial pressure that yields a numerical value, measuring both opening and closing pressures [8]. However, LP is not always recommended or obtainable as a result of body habitus, clinical condition, or anatomical variations [9-10].

In the 1960’s, Lundberg initiated the advent of intraventricular pressure monitoring [11], and the cannulation of the ventricle became the gold standard for measuring ICP. However, ventriculostomy has the risks of malposition, hemorrhage, and infection as potentially associated adverse events [12-13]. As a result of this, alternative ICP monitoring modalities have developed over the last few decades [14-15]. Subdural devices based on saline-filled transducers and electrical impedance came about in the 1980’s [16] but grew out of favor because of problems associated with ICP measurement drift over time. In cases of severe traumatic brain injury with significant edema and small ventricles, ventricular catheterization becomes a challenge. Extraventricular devices, such as intraparenchymal microsensors, were developed to give clinicians an alternate way of monitoring ICP. Intraparenchymal monitors play a crucial role in providing readings very similar to those produced via ventricular catheterization. They have a lower profile and pose fewer risks to the patient [8].

More recently, an emphasis has been placed on monitoring other parameters aside from intracranial pressure. Brain oxygen tension and temperature, biochemical analysis, and invasive and non-invasive electroencephalography are now being utilized to guide further treatment in the pediatric neuro-ICU setting [15].

## Review

### Intracranial monitoring and pediatric age

Reported animal studies have suggested that age plays a role in the response of the brain to a traumatic event. Younger animals tend to display a longer decrease in cerebral blood flow and hypotension than older-aged animals in response to a diffuse traumatic brain injury [14, 17-18]. Inflammation also plays a significant role in pediatric brain injury; levels of interleukin-6 and interleukin-10 become elevated in the cerebrospinal fluid (CSF) of infants and children in response to a severe traumatic brain injury (TBI) [19-20]. There is also an age-dependent production of interleukin-1, and children younger than four years old tend to display higher concentrations of this inflammatory mediator [19-20].

Within the first few months of life, some children, both premature and term infants, experience germinal matrix hemorrhages and choroid plexus hemorrhages, respectively [1, 21]. One of the most basic clinical methods of intracranial pressure monitoring in this age group, although imprecise and without a numerical value, is palpation of the open anterior fontanel and cranial sutures, as well as serial head circumference measurements [22-23]. The open anterior fontanel serves as a surrogate for indirectly assessing ICP, and a splaying of the cranial sutures beyond what is felt to be within the normal range is also of subjective clinical value [24]. Increased daily measurements of the head circumference, coupled with a full and tense fontanel, as well as splayed sutures may suggest an increased intracranial pressure. Kaiser and Whitelaw simultaneously assessed intracranial pressure in neonates by palpation of the anterior fontanel and direct measurement via cerebrospinal fluid compartment cannulation. The mean pressure was found to be significantly different between “soft” (5.4 mmHg) and “tense” (14 mmHg) fontanels. Despite these differences, there was considerable overlap [25]. Hence, the full reliability of this surrogate for intracranial pressure monitoring is cautioned. 

There is a lack of consensus on the "normal value" of ICP in children. Such parameters have been well established to some degree in the adult population. However, establishing such values is more challenging in the pediatric population, given the rapidly changing intracranial physiology as a child grows. Knowledge of the accepted intracranial pressure values based on age differences is critical to the clinician during interpretation of invasive monitoring, i.e., an ICP that would otherwise be interpreted as “benign” in a teenager may be severely pathological in a three-year-old child. Between the ages of four and 16, the highest acceptable value for ICP is agreed upon to be 12.9 mmHg. An upper limit of 5 mmHg is typically regarded as normal in a mechanically ventilated infant after a TBI. A slightly older child in a similar clinical scenario should have an ICP no higher than 10 mmHg [8, 26-27]. Mehta and colleagues examined the relationship between intracranial pressure and cerebral perfusion pressure. They concluded that children with a traumatic brain injury subsequently having an ‘unfavorable outcome’ had more frequent recordings of a cerebral perfusion pressure < 45 mmHg in comparison to children with a ‘favorable outcome’. This study suggested a target cerebral perfusion pressure of at least 45 mmHg to avoid brain ischemia [28].

### Indications for multimodal monitoring

The mean objective in placing a device to monitor a specific parameter of interest is the early detection of abnormal processes to avoid secondary brain insults. Insults from decreased substrate delivery, hypoxia, hypoglycemia, hypotension, hypovolemia, worsening or development of cerebral edema and intracranial hypertension, hyperglycemia, hypercapnia, cellular toxicity, hyperglycemia, hyperoxemia, increased metabolism, fever, seizure, negative nitrogen balance, systemic derangement, infection, venous thrombosis, drugs, or multi-factorial processes. With monitoring, neuro-intensivists can individualize therapeutic goals. Optimizing treatment dosing and timing reduces potential adverse effects of an intervention, hopefully translating to improved outcome.  Also, monitoring enables the understanding of the pathophysiology regarding complex processes occurring simultaneously in the injured brain. Intracranial pressure monitoring alone may not detect other metabolic dysfunctions leading to secondary injury. Hence, there is a role for multimodal monitoring in the insulted pediatric brain [29]. It should be kept in mind that the majority of devices on the market are made to accommodate an adult-sized skull thickness. A need exists to specifically develop monitoring devices that will fit appropriately in order to obtain the most accurate readings.

Lumbar Puncture (LP)

First described in 1891 by the German physician, Heinrich Quincke, the lumbar puncture (LP) is one of the oldest methods of indirectly measuring intracranial pressure [30]. Reliance on the manometer reading for opening pressure rests on whether one believes that the pressure gradient between the intracranial cavity and lumbar cistern is essentially negligible [31-32]. After an intracerebral insult, cerebrospinal fluid does not circulate freely as it would normally; CSF volume may actually be low as a result of compensatory mechanisms instituted by the brain to accommodate a new inhabitant, whether it is blood or edema. There has always been a question of whether there is one uniform intracranial pressure, or whether ICP is compartmentalized [12, 33]. A non-communicating ventricular system with the spinal theca may yield an unreliable “ICP value” obtained via a lumbar puncture [34-35]. Lumbar punctures can be challenging in the setting of a neonate or a compartmentalized intrathecal space. It should also be kept in mind that this maneuver has the potential to result in tonsillar herniation in some patients [34-35]. As with placement of a lumbar drain, it is of vital importance prior to performing a lumbar puncture to ensure that there is no significant supratentorial mass effect and that there are radiographically open basal cisterns.

Lumbar Drain (LD)

Studies have shown efficacy in the use of lumbar drains as a means for controlling increased intracranial pressure. However, data is lacking in regards to the use of continuous ICP monitoring utilizing a lumbar drain in the neurocritical care patient population after sustaining a brain injury [31]. After a significant intracerebral insult, whether it be from significant head trauma or spontaneous subarachnoid hemorrhage, an external ventricular drain (EVD) is often utilized for both diagnostic and therapeutic purposes. Subsequent placement of a lumbar drain in cases of medically refractory intracranial hypertension, even after an EVD has been placed, has shown a significant reduction in ICP measurements [36-39]. The most feared complication in regards to placement of a lumbar drain in the setting of increased intracranial pressure is the potential for downward tonsillar herniation [40]. As with LP, this can occur as a result of over-drainage through the lumbar cistern, which can be potentiated by a high cranial to a caudal pressure gradient. This highlights the fact that controlled use of a lumbar drain for both therapeutic and diagnostic purposes in the pediatric brain injury population is best instituted with basal cisterns radiographically open and no evidence of a significantly-sized lesion causing mass effect and midline shifting up structures. It may also be a safer option to place an external ventricular drain prior to institution of a lumbar drain [41]

External Ventricular Drain (EVD)

Claude-Nicholas Le Cat performed the first documented external ventricular drain (EVD) in 1744 for congenital hydrocephalus. Evolution of this technique has taken place over time [42]. EVDs have become the gold standard to measure intracranial pressure [43]. In addition to being a diagnostic tool for measuring ICP, EVDs are also used for therapeutic purposes [8]. Common indications for placing an EVD in the pediatric population include traumatic brain injury, acute hydrocephalus from various causes, and ventriculoperitoneal shunt failure [44]. Complications with the placement of an external ventricular drain include misplacement (which can lead to neurological deficits), infection, hemorrhage, and malfunction necessitating replacement [44]. Rates of secondary EVD-related infection in regards to placement in the pediatric intensive care unit as opposed to the operating room (OR) have not been assessed [44]. Ngo, et al. specifically looked at rates of EVD-related complications in pediatrics; in this study, 66 patient charts were reviewed, and 96 EVD’s were placed [44]. The overall total complication rate was 26%. Catheter infection occurred in 9.4%, catheter misplacement was seen in 6.3%, and hemorrhage was seen as a complication in 4.2% of insertions. Adult literature, as well as the results obtained from Ngo, et al., agree that EVDs inserted at the bedside with sterile technique were not associated with higher complications rates when compared to EVDs inserted in the operating room [44-45]. Patients with traumatic brain injury and small ventricular size have not shown an increased risk of EVD misplacement versus pediatric patients with hydrocephalus. Placement of adult size trauma EVDs in small pediatric ventricles may also yield false values, given the collapse of the ventricular walls around the device.

Transcranial Doppler (TCD)

Transcranial Doppler (TCD) has been a vital non-invasive tool in the repertoire of the neurocritical care team allowing real-time monitoring of physiological changes in the blood flow velocity. In the adult population, TCDs are routinely used to assess for vasospasm following spontaneous subarachnoid hemorrhage to detect velocity changes of blood flow consistent with an elevation of ICP [46]. In the pediatric brain trauma population, it has been suggested that TCD could be used as an initial monitoring modality to indirectly measure ICPs and cerebral perfusion pressure (CPP). TCD has shown to have a 94% sensitivity (possibility of screening patients for potentially elevated ICP) and a 95% negative predictive value (ability to identify patients with a normal ICP) [47]. Furthermore, TCD can be performed within the first few minutes of a patient arriving at the hospital if there is a concern for increased intracranial pressure. On the contrary, Figaji, et al. concluded that the pulsatility index of TCDs is not a consistent representation of intracranial pressure in children with significant TBIs. In their study, they only found a minor association between the pulsatility index and the ICP [48]. Further studies are necessary to help establish the relationship between transcranial Doppler pulsatility index and CPP.

TCDs have also been used to evaluate cerebral autoregulation (AR); when the mean arterial pressure (MAP) elevates, constriction of the intracranial vasculature takes place. Conversely, the intracranial vasculature dilates as the mean arterial pressure decreases. This physiological event remains constant over a MAP range from 50 to 150 mmHg in the uninjured brain of an adult [43]. A formula was created to measure if the autoregulatory response is appropriate utilizing the transcranial doppler. The formula is ARI = %Δ eCVR/%Δ MAP (ARI = autoregulation index, eCVR = estimated cerebrovascular resistance, MAP = mean arterial pressure). An autoregulatory index ≥ 0.4 is regarded as normal. Autoregulatory impairment has been observed in as many as two-thirds of adults and 40% of the pediatric population with significant traumatic brain injuries [49-52]. It has also been related to an overall poor outcome in both pediatric and adult traumatic brain injury patients. However, it is not clear if autoregulatory impairment is itself an isolated variable leading to a significantly decreased clinical outcome [53-55].

Parenchymal Intracranial Pressure Monitoring

The most widely used and commercially available device for direct parenchymal ICP monitoring is named the Camino (Integra Life Sciences, New Jersey). The monitor itself is a fiber optic lead inserted directly into the brain parenchyma to a predetermined depth. It is designed for rapid placement in the neuro-ICU setting to provide an immediate ICP value and associated waveform. Intraparenchymal ICP monitoring devices are safe with a rather low complication rate [56]. Procedure-related complications, such as brain contusion, epidural hematoma, and catheter disconnection have been reported [56]. As a disadvantage, these devices are not MRI-compatible. This limits its use in children where MRI is often favored over repeated CT scans, as MRI yields prognostic information and also limits exposure to radiation from CT scans. Besides, ICP “drift” has been a well-documented limitation, whereby the values obtained may lose reliability with time [57]. In small children, with thinner skulls compared to adults, non-bolted devices are often necessary, given the inability of the skull to support the bolted version. The depth of the bolt itself has the potential to exceed the skull thickness, putting the underlying brain at risk during placement. Tunneled versions of such intraparenchymal monitors have been designed, excluding the need for a bolt, but implying a greater likelihood of malposition due to the lack of a “guiding system” for insertion.

Continuous Brain Tissue Oxygen Monitoring

Brain tissue oxygenation (PbtO2) has been a more novel target parameter that has been studied in the adult and pediatric population in regards to TBI as well as subarachnoid hemorrhage-induced vasospasm. PbtO2 monitoring is utilized via an invasive method, whereby an electrode is inserted into a specific area of interest in the brain parenchyma. PbtO2 is measured by the utilization of the Licox device (Integra Life Sciences, New Jersey). Licox is a triple lumen device allowing simultaneous monitoring of ICP, PbtO2, and brain temperature. In the head-injured population, it has been shown that brain temperature exceeds systemic temperature and may increase on average by 2.0 degrees F (1.1 degrees C) above the core body temperature [58]. As we aim to avoid hyperpyrexia in the brain-injured patient, establishment of target core brain temperatures in the pediatric population needs to be established.

The Licox is placed at Kocher’s point unless placement in that region would entail insertion into a known hematoma or ischemic region, which may ultimately yield false values. The catheter membrane allows oxygen to diffuse across it, and this subsequently generates a voltage. The degree of oxygenation at the specific electrode site is proportional to the generated voltage. Still, the physiological representation of the brain-tissue-oxygen-tension value continues to be under investigation [59]. It has been reported that diminished oxygenation of brain tissue is associated with a worse prognosis [17, 60-62]. However, there is not enough data available to support that PbtO2 in children with cerebrovascular accidents would aide in the prevention of progressive ischemic events and ultimately improved outcome [17]. Narotam, et al. were able to show that a brain tissue oxygenation-directed protocol reduced the mortality rate after major traumatic brain injury in a patient population with a mean age of 35 years. It also resulted in an improved six-month clinical outcome over the standard ICP/CPP-directed therapy at the authors’ institution [16]. Figaji and colleagues demonstrated that reduced PbtO2 in children after sustaining a severe traumatic brain injury appears to be an independent factor associated with poor outcomes [63-64]. PbtO2 values <10 mmHg had a significant association with an irreversible brain insult. The most recent consensus on pediatric TBI guidelines made a Level III recommendation to give consideration to maintaining a PbtO2 ≥ 10 mmH2O [65]. Stippler and colleagues studied the utilization and outcomes of PbtO2 monitoring in children with severe TBI’s, with simultaneous ICP and CPP recording. Results demonstrated some instances of unfavorable clinical results with altered ICP and CPP values, despite normal PbO2 values [66].

The interpretation of PbtO2 monitoring in pediatric TBI is a complex issue. Effective and efficacious PbtO2-driven management protocols are yet to be established.

Jugular Venous Oxygen Saturation Monitoring

Continuous measurement of jugular venous oxygen saturation (SjvO2) is typically evaluated using a fiberoptic catheter positioned in the internal jugular bulb. SjvO2 has been utilized to detect cerebral ischemia after brain injury [67-68]. Perez and colleagues were able to show that two or more measurements of SjvO2 at 55% or less were associated with a significantly poor neurological outcome in children with severe brain injuries [69]. Despite the associations of low SjvO2 with adverse neurological sequels, intermittent SjvO2 monitoring has not been shown to alter substantially the management of severely brain-injured individuals amongst clinicians [18]. A high incidence of erroneous readings from SjvO2 monitoring has been reported, which limits the reliability when making crucial management decisions [70].

Cerebral Microdialysis

Cerebral microdialysis is a tool that can be used to improve the understanding of cerebral energy metabolism [71]. Traditionally used as a research tool, microdialysis is now being brought into the neurointensive care setting with the main goal of identifying early markers of ischemia and cell damage [72]. The system uses a thin, fenestrated, double-lumen catheter inserted into the brain interstitium. It does evaluate markers of cell metabolism (lactate, pyruvate, and glucose), neurotransmitters (glutamate), or tissue damage markers (glycerol) that correlate with a level of outcome [73]. A dialysate is infused into the catheter at a slow rate. There is a semipermeable membrane through which small molecules diffuse across from the interstitium into the dialysate. The specimen is then obtained and sent for analysis at desired intervals.

One of the downsides to this modality is that the data collected is not available for the clinicians in real-time and the neurochemical data that is observed appears to reflect the area where the probe was inserted.  Also, the insertion of the catheter alone causes local microtrauma, which can induce an inflammatory reaction. For this reason, sample collection analysis is often postponed by one to two hours [71].

Tolias, et al. reported the first use of microdialysis in children with TBI. This manuscript focused on neurotransmitter levels as well as that of other amino acids [74]. The authors concluded that differences existed regarding excitatory neurotransmitters in the pediatric population relative to prior descriptions in adult literature. Normal limit values for a multitude of small molecules have been created in the adult population but are not determined in children [75].

Electroencephalography (EEG)

Continuous electroencephalography (cEEG) has been used in the pediatric brain-injured population to identify clinical and subclinical seizures and encephalopathy [76]. The traditional method of using cEEG is via scalp electrodes, which often leads to records contaminated by muscle artifact and lack of precise spatial resolution [77]. As a result of this, a novel “mini depth” multi-contact electrode has been developed. Specialized multi-contact electrodes are placed into the cerebral cortex via a burr hole made at the bedside and corticography is recorded. It has been found that through intracortical encephalography (ICE) recordings, many seizures were displayed that were not readily identified on the traditional scalp electrode EEG. Some authors hypothesize that multifocal, poorly synchronized “mini-seizures” may, in fact, contribute to global cortical dysfunction seen after traumatic brain injury. The ultimate goal of using ICE in the future is to develop utilization of an EEG-alarm system, aiding in the prevention of secondary brain injury [77].

Near-infrared Reflective Spectroscopy (NIRS)

Transcranial near-infrared reflective spectroscopy (NIRS) is a non-invasive modality used for monitoring regional intracerebral oxygen saturation. The most commonly utilized commercially available system is the EQUANOX (Nonin Medical, Minneapolis, MN). The device is a lightweight sensor that attaches to the frontal scalp region of the patient. The system can be mounted on a pole and is portable. Specifically, the NIRS looks at brain oxygen delivery and utilization [65, 78]. NIRS is also used for detecting intracerebral blood volume changes [79]. Kamfl, et al. studied the use of NIRS in adults in the neurointensive care unit and were able to show that NIRS correlated with clinical signs of hypoperfusion and decreased oxygen saturation [80]. The authors were also able to demonstrate a significant difference in regional intracerebral oxygenation between patients with both normal and elevated intracranial pressure, showing that patients with increased ICP, greater than 25 mmHg, had lower regional intracerebral oxygen saturation [79-80]. The use of NIRS has been also applied in the setting of assessment for bifrontal regional cortical oxygen saturation in coronary bypass patients. NIRS has demonstrated that this adult patient population had low regional intracerebral oxygen saturation and was linked with cognitive dysfunction, an extended course, and perioperative strokes. 

In the setting of the neonatal ICU, NIRS has been used to determine cerebral oxygenation in sick children and has been able to provide important information regarding regional tissue perfusion [78]. However, to date, there have been no studies looking specifically at the use of NIRS with other intracranial pressure/oxygenation monitoring devices. Until such studies are performed, it will be difficult to draw conclusions as to the potential benefit of its use in the pediatric brain-injured population.

Biomarkers      

At the time of a brain insult, support cells and neurons undergo structural damage; this ultimately leads to extravasation of certain proteins into the blood, cerebrospinal fluid, and extracellular matrix. These biomarkers can then be utilized and interpreted in such a way that they may hold predictive value in the degree of parenchymal brain injury [81]. Investigations into various biomarkers have been utilized as an adjunct to predicting neurological outcomes in the pediatric brain-injured population. Ubiquitin c-terminal hydrolase (UCH-L1), alpha II-spectrin breakdown product 145 kDa (SPDP 145), glial fibrillary acidic protein (GFAP), neuron-specific enolase (NSE), S100B, and myelin basic protein (MBP) have specifically been investigated. In the setting of TBI, the biomarkers’ concentrations have been analyzed. In a study by Berger and colleagues, higher concentrations of NSE, S100B, and MBP were associated with worse outcomes [82]. Increases in ubiquitin c-terminal hydrolase (UCH-L1) and alpha II-spectrin breakdown product 145 kDa (SPDP 145) have specifically been seen in pediatric subjects with moderate and severe TBI. UCH-L1 and SPDP 145 have also been found to have a stronger correlation with the Glasgow Outcome Scale than NSE, S100B, and MBP [82]. GFAP is not found outside the central nervous system and is thus a more specific biomarker. GFAP has also been found to be significantly elevated in pediatric brain-injured patients [83]. Correlation between various biomarkers to different subtypes of brain injuries can be tested to provide a stronger and more specific prognostic value to particular markers.

## Conclusions

Monitoring intracranial pressure is a vital component of the management of the brain-injured pediatric patient. Other types of potentially useful information, aside from ICP, are now being monitored to further aid in the earlier detection of deleterious secondary brain injury. To date, there has been very scant literature correlating traditional ICP monitoring to other modalities that yield different types of diagnostic information.  It is warranted to design a study collecting intracranial pressure values derived from an EVD and/or an intraparenchymal pressure monitor and correlate these values to the diagnostic information derived from other non-invasive modalities. There is a need for non-invasive, or at least less invasive ways of monitoring, that can be relied upon. In this regard, normal values of non-invasive monitoring need first to be established in the pediatric population prior to comparing them to that of the injured pediatric brain. In the future, large data analysis and visualization of complex interrelated physiologic signals may yield the safest, least invasive methods of deriving useful information for the optimal care of pediatric TBI patients.
